# Probiotic potentials of lactic acid bacteria isolated from Egyptian fermented food

**DOI:** 10.1038/s41598-023-43752-0

**Published:** 2023-10-03

**Authors:** Fatma I. Abdel Tawab, Menna H. Abd Elkadr, Amany M. Sultan, Ehdaa O. Hamed, Ayatollah S. El-Zayat, Marwa N. Ahmed

**Affiliations:** 1 Oil Crops Biotechnology Lab, Agricultural Genetic Engineering Institute, Agricultural Research Center, Giza, Egypt; 2 Microbiology Lab, Research Park, Faculty of Agriculture, Cairo University, Giza, Egypt; 3 Department of Biochemistry, Toxicology Unit, Animal Health Research Institute, Agricultural Research Center, Giza, Egypt; 4 Department of Microbiology, Faculty of Agriculture, Cairo University, El-Gamaa Street, Giza, 12613 Egypt

**Keywords:** Biotechnology, Microbiology

## Abstract

Lactic acid bacteria (LAB) are of major concern due to their health benefits. Fermented food products comprise variable LAB demonstrating probiotic properties. Discovering and evaluating new probiotics in fermented food products poses a global economic and health importance. Therefore, the present work aimed to investigate and evaluate the probiotic potentials of LAB strains isolated from Egyptian fermented food. In this study, we isolated and functionally characterized 100 bacterial strains isolated from different Egyptian fermented food sources as probiotics. Only four LAB strains amongst the isolated LAB showed probiotic attributes and are considered to be safe for their implementation as feed or dietary supplements. Additionally, they were shown to exert antimicrobial activities against pathogenic bacteria and anticancer effects against the colon cancer cell line Caco-2. The *Enterococcus massiliensis* IS06 strain was exclusively reported in this study as a probiotic strain with high antimicrobial, antioxidant, and anti-colon cancer activity. Hitherto, few studies have focused on elucidating the impact of probiotic supplementation in vivo*.* Therefore*,* in the current study, the safety of the four strains was tested in vivo through the supplementation of rats with potential probiotic strains for 21 days. The results revealed that probiotic bacterial supplementation in rats did not adversely affect the general health of rats. The *Lactiplantibacillus plantarum* IS07 strain significantly increased the growth performance of rats. Furthermore, the four strains exhibited increased levels of antioxidants such as superoxide dismutase and glutathione in vivo. Consistently, all strains also showed high antioxidant activity of the superoxide dismutase enzyme in vitro. Overall, these findings demonstrated that these isolated potential probiotics harbor desirable characteristics and can be applied widely as feed additives for animals or as dietary supplements for humans to exert their health benefits and combat serious diseases.

## Introduction

Fermented foods have been a substantial part of the human diet for several years^1^. Additionally, fermented food constituted of plants, meat, or milk can be maintained for a longer time than fresh raw food^2^. Fermented foods comprise a good reservoir of probiotic bacteria. Probiotics are strains of microorganisms that exert positive health effects when administered to humans and animals. Probiotics are mainly lactic acid bacteria (LAB) belonging to the genera *Lactobacillus* and *Bifidobacterium*^3^. Other genera can also be used as probiotics, such as *Bacillus*, *Enterococcus*, *Pediococcus*, and several yeasts^4^. LAB are mostly gram-positive, anaerobic rods that are derived from the human gastrointestinal tract^5^.

LAB were originally isolated from fermented food in single or mixed cultures but are the best candidate for enhancing the fermentation process^6^. They also have the capacity to survive during the fermentation process^7^. LAB isolated from fermented food with the capacity to improve digestive health are considered probiotics^8^. They are typically used as potential microbe-containing food supplements and are regarded as a crucial functional food constituent^9^. In addition to fermented food, LAB can exist in a variety of habitats, such as gastrointestinal tracts (GIT), oral cavities, human and animal vaginal tracts, silages, and composts^10^. LAB confer a variety of advantageous health benefits to the host, including improving the digestion process^11^, controlling inflammatory bowel diseases^12^, maintaining the healthy balance of the gut microbiota^13^, and modulating immune function^14^. *Lacticaseibacillus rhamnosus*, a LAB strain previously isolated from human breast milk has been reported to modulate the intestinal microbiota and immune system^15^. Additionally, *Lactobacillus plantarum* supplementation has been shown to stimulate lead detoxification by increasing the rate of lead excretion in the animal feces^15^. Furthermore, some LAB strains possess anticancer and antidiabetic effects^16,17^. The exopolysaccharides of *Lactobacillus* strains have been proved to have anticancer, antidiabetic, antioxidant, antimicrobial and immunostimulatory effects^18–22^. Therefore, LAB have been considered to be the best candidate probiotics.

One of the most important features of LAB is the production of significant metabolites such as lactic acid, organic acids, antioxidants, and antimicrobial compounds^23^. These metabolites play an important role in maintaining and modulating the gut microbiota. LAB supplementation in animals enhances growth rate performance, leading to higher meat production and lower rates of disease incidence^24^. Consequently, probiotics have been widely used as feed additives and as proper alternatives to antibiotics. For example, probiotic supplementation in poultry can improve the immune response and growth rate^25^. Other animals, such as pigs^26^, silver foxes^27^, ostriches^28^, and raccoon dogs^27^, have also shown similar outcomes. Currently, investigating new probiotics, particularly from newly discovered species, has become an important practice^29^.

LAB should be assessed and evaluated as potential probiotic candidates and must fulfill all the necessary quality assurance criteria for probiotic selection^30^. These criteria include antibiotic resistance, showing no virulence or toxigenic activity, tolerance to GIT conditions, adherence capacity to intestinal epithelial cells, and autoaggregation and coaggregation activities^31^. In addition, probiotics must maintain their stability and vitality inside the host and during the food processing and preservation process. Therefore, the main purpose of the current study is to isolate and assess LAB from different fermented food products in vitro and in vivo to explore new safe probiotics with promising antimicrobial, antioxidant, and anticancer applications.

## Materials and methods

### Sample collection, LAB isolation and culture conditions

**Figure 1:**
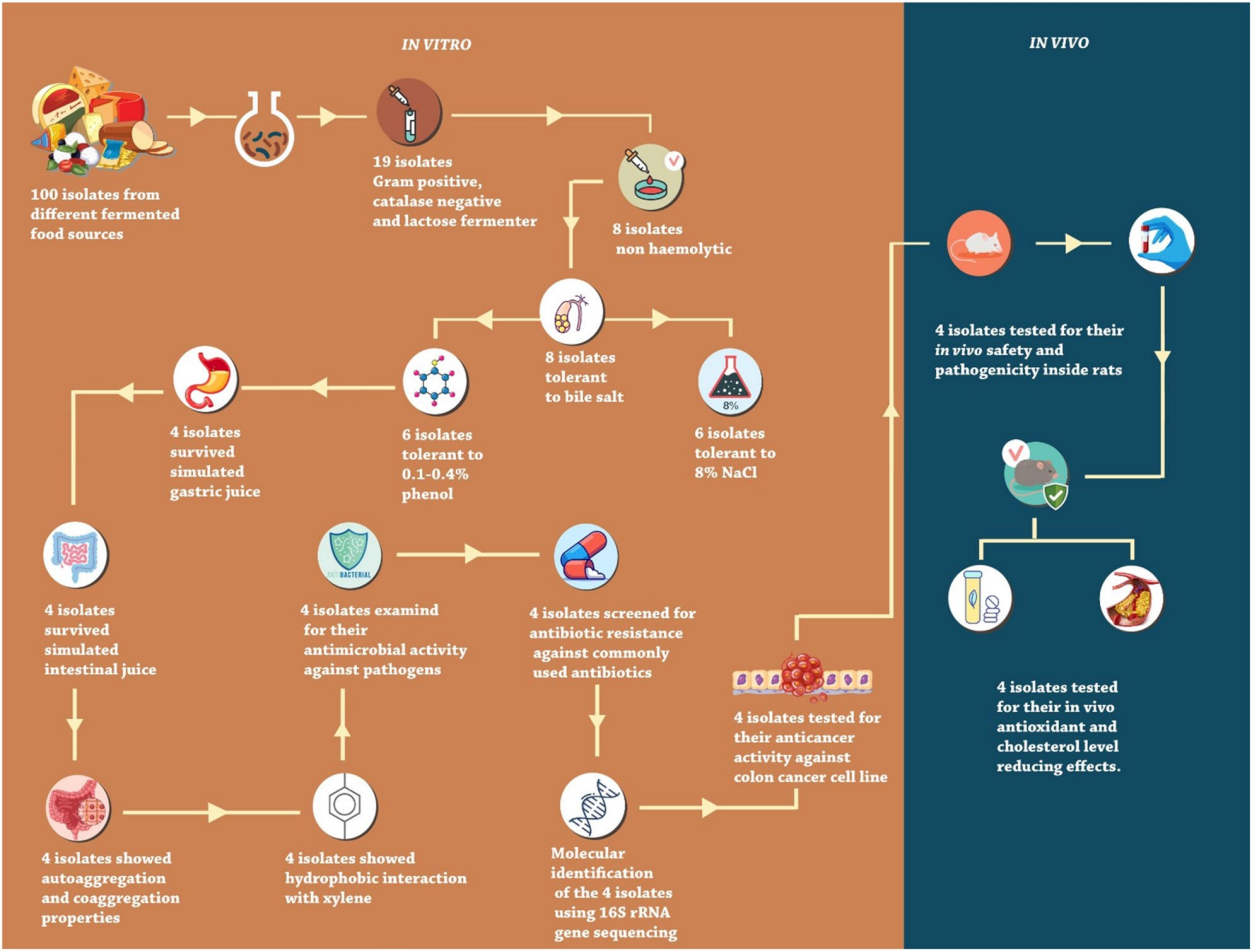
Experimental workflow for the isolation and assessment of LAB as probiotics from different fermented food sources.

Eight fermented food products were collected from markets in Egypt to isolate potential probiotic LAB. These eight products included plant-based luncheon meat, soy sausage, corned beef, soybean, pickles, kareish cheese, rayeb milk, and full-fat milk. All these samples were screened for the presence of probiotic LAB. The dilution-plate method was applied to isolate LAB on de Man, Rogosa, and Sharpe (MRS) agar at 37 °C for 24–48 h. The colonies grown on MRS media were collected and subjected to biochemical and morphological analysis. Gram-positive, catalase-negative, and lactose-fermenting bacteria were only selected and regarded as LAB candidates. All LAB isolates were maintained in 20% glycerol at − 20 °C for further analysis (Fig. 1).

### Bacterial strains

The indicator pathogenic bacteria used in this study were purchased from American-type culture collection (ATCC). These pathogens include *Escherichia coli* O157:H7 ATCC 700,728, *Pseudomonas aeruginosa* ATCC 35,032, *Salmonella enterica* subsp. *enterica* serovar Typhimurium ATCC 14,028, *Staphylococcus aureus* ATCC 25,923, Methicillin-resistant *Staphylococcus aureus* (MRSA) ATCC 43,300, *Bacillus cereus* ATCC 33,018, and *Listeria monocytogenes* ATCC 19,115. All pathogenic strains were cultured and propagated on a tryptone soya yeast extract broth (TSYEB) medium for coaggregation and antagonistic activity assays.

### Safety assessment of the LAB strains

#### Hemolysis assay

The hemolytic activity of the selected LAB strains was determined according to Maragkoudakis et al*.*^32^. LAB strains were grown overnight in MRS broth. Following incubation, an aliquot of each overnight culture was streaked onto a blood agar base (Oxoid, UK) containing sheep blood 5% (v/v). The plates were incubated at 37 °C for 24 h. The grown LAB strains were screened for α-, β-, or γ-hemolysis. LAB strains that showed no blood or γ-hemolysis were considered safe.

#### Antibiotic susceptibility assay

The LAB isolates were tested for their antibiotic susceptibility against commonly used antibiotics using a Kirby-Bauer disc diffusion assay^35^. The antibiotics applied in this study were ceftriaxone (30 µg), penicillin G (2 units), ampicillin (10 µg), gentamicin (10 µg), oxacillin (1 µg), streptomycin (10 µg), tetracycline (30 µg), vancomycin (30 µg), ciprofloxacin (5 µg), chloramphenicol (30 µg), erythromycin (10 µg), and kanamycin (30 µg). MRS plates inoculated with LAB isolates and with antibiotic discs placed on the surface of agar were incubated at 37 °C for 24 h. The diameters of the inhibition zones were measured, and the results were interpreted according to the guidelines of the Clinical and Laboratory Standards (CLSI 2014).

### Probiotic evaluation of the LAB strains

#### Bile salt tolerance

The ability of LAB strains to survive in the presence of 0.3% bile salt was assessed. Overnight LAB cultures (1% v/v) were inoculated into MRS broth supplemented with 0.3% bile salt, followed by subsequent incubation. The viability of LAB strains was recorded at 0 and 6 h of incubation by determining the colony-forming unit (CFU) counts of strains on MRS agar.

#### NaCl and phenol tolerance

The tolerance of LAB strains to NaCl was determined by inoculating the overnight cultures of LAB strains (1% v/v) into MRS broth containing 8% NaCl. Following incubation at 37 °C for 6 h, the optical density of the grown LAB cultures at a wavelength of 600 nm (OD_600nm_) was measured using a spectrophotometer (Metash UV-800, Shanghai, China) to assess their viability.

Similarly, the ability of LAB strains to tolerate high concentrations of phenol was assessed. Overnight cultures of LAB strains (1% v/v) were inoculated into MRS broth containing increasing concentrations of phenol (0.1–0.4%)^33^. The inoculated MRS broth tubes were incubated at 37 °C for 24 h. The viability of the strains was determined by measuring the OD_600nm_ of grown LAB strains using a spectrophotometer.

#### Simulated gastric and intestinal juice survivability

The capacity of LAB strains to survive in the simulated human digestive system was investigated according to the method described by Botta et al.^34^. Briefly, overnight LAB cultures were centrifuged and resuspended in simulated gastric juice containing pepsin (pH 2)^35^. The inoculated flasks were subsequently incubated at 37 °C for 4 h in an orbital shaker at a speed of 200 rpm. The CFU counts of each selected LAB strain were determined at 0 and 4 h incubation by plating into MRS agar. The cells grown in simulated gastric juice were collected by centrifugation and washed in phosphate-buffered saline (PBS). The cells were then resuspended in simulated intestinal juice containing pancreatin (pH 6.8)^36^. Immediately after incubation at 37 °C for 4 h, the viability of LAB strains was recorded by determining the CFU counts as described earlier.

#### Autoaggregation and coaggregation assays

The autoaggregation capability of LAB strains was determined using the method described by Polak-Berecka et al.^37^ with some modifications. In short, the overnight LAB cultures were centrifuged and washed with PBS. The harvested cells were resuspended in 2 mL of PBS, and the OD_600nm_ was adjusted to 0.25 ± 0.02. The bacterial suspension was incubated at 37 °C for 2 h. The absorbance of the bacterial suspension was measured at 600 nm using a spectrophotometer at 0 and 2 h time points. The autoaggregation activity was determined according to the following formula:

(1 − A_0h_/A_2h_) *100.

Where A_2h_ denotes the absorbance at t = 2 h and A_0h_ denotes absorbance at t = 0 h.

For the determination of the coaggregation capability of LAB strains, the bacterial suspensions were similarly prepared as described above. Two milliliters of each LAB strain and pathogen were mixed and incubated at 37 °C for 2 h. Control tubes contained 4 mL of each LAB strain and each pathogen. The absorbance of the mixed bacterial suspensions was measured at 600 nm after 2 h of incubation and compared to that of the control suspensions for probiotic and pathogen strains. The coaggregation activity was calculated as follows^38^: [1 − A_mix_/ (A_LAB_ + A_pathogen_)/2]*100.

Where A_mix_ denotes the absorbance of mixed suspensions, A_LAB_ denotes the absorbance of control tubes containing LAB strains, and A_pathogen_ denotes the absorbance of control tubes containing pathogens.

#### Cell hydrophobicity assay

The LAB cell hydrophobicity was assayed by measuring the percentage of bacterial adhesion to hydrocarbons^39^. Bacterial cells and xylene mixtures were prepared by mixing 2 mL of each LAB strain suspended in PBS at 10^8^ CFU/mL with 2 mL of xylene. The mixture was then vortexed, and the OD_600_ of the aqueous phase was measured with a spectrophotometer. The affinity of LAB strains to xylene was calculated using the following equation: [(A_0_ − A_30min)/_A_0_] *100.

#### Antagonistic activity of LAB strains

The antimicrobial activity of LAB strains was determined using an agar-plug diffusion assay^40^. The LAB strains were streaked onto MRS agar and incubated at 37 °C for 5 days to allow the isolates to secrete their metabolites. After incubation, an agar plug was cut aseptically and placed on the surface of Mueller Hinton agar (Biolife, Italia) inoculated with the pathogen. The plates were incubated at 37 °C for 24 h. The antimicrobial activity was determined in terms of the inhibition zone diameters (mm) surrounding the agar plugs of each LAB strain.

### Molecular identification of LAB strains

LAB strains were identified using 16S rRNA amplification and sequencing with universal forward (5′-AGAGTTTGATCMTGGCTCAG-3′) and reverse (5′-AAGGAGGTGATCCAGCC-3′) primers. Then, the PCR reactions were conducted using a previously described protocol^41^. The amplified PCR products were visualized using a gel electrophoresis and gel documentation system (Bio-Rad, USA) and then purified and sequenced. The 16S rRNA sequences were aligned with the bacterial sequences in the NCBI database using the basic local alignment search tool (BLAST). Phylogenetic analysis of the identified LAB strains was performed using the neighbor-joining method. The tree was constructed using the maximum composite likelihood method.

### Cytotoxicity assessment of LAB strains against colon cancer cell line

The LAB strains were allowed to grow for five days in MRS broth. Cell-free supernatants were obtained by centrifugation of LAB cultures at 6000 rpm for 10 min (Hettich, Germany). The cell proliferation activity of LAB strains was measured against the human epithelial colorectal adenocarcinoma (Caco-2) cell line using the MTT (3-[4,5-dimethylthiazole-2-yl]-2,5-diphenyltetrazolium bromide) cell viability assay. The Caco-2 cell line was provided from ATCC, USA, and routinely cultured in RPMI-1640 medium (Roswell Park Memorial Institute). This medium was supplemented with 10% fetal bovine serum (FBS), 2 mM L-glutamine, 100 units/ml penicillin G sodium, 100 units/ml streptomycin sulfate, and 250 ng/ml amphotericin B (Nuaillé, France). Cells were kept at subconfluency at 37 °C in humidified air containing 5% CO_2_. For subculturing, monolayer cells were harvested after trypsin/EDTA treatment at 37 °C. Cells were used when confluence reached 75%. All cell culture materials were obtained from Cambrex Bioscience (Copenhagen, Denmark). The supernatants of LAB strains were initially diluted in the medium to obtain the desired concentrations for the assay. Cells (5 × 10^4^ cells/well) in RPMI-1640 medium were plated in a flat-bottom 96-well microplate and treated with 20 µL of different concentrations of the LAB supernatants for 48 h at 37 °C in a humidified 5% CO_2_ atmosphere, except for the 20% v/v concentration, which was treated with 40 µL of the tested sample over 160 µL of the cells. The tested concentrations were 10, 5, 2.5, 1.25, and 0.625% (v/v). After incubation, the medium was removed, and an aliquot of 40 µL MTT solution/well was added and incubated for an additional 4 h. MTT crystals were solubilized by adding 180 µl of acidified isopropanol/well, and the plate was shaken at room temperature, followed by photometric determination of the absorbance at 570 nm using a microplate ELISA reader (FLUOstar OPTIMA, BMG LABTECH GmbH, Ortenberg, Germany)^42^. Four repeats were performed for each concentration, and the average was calculated. Data were expressed as the percentage of relative viability compared with the untreated cells, with cytotoxicity indicated by < 100% relative viability. The percentage of relative viability was calculated using the following equation:

(Absorbance of treated cells/Absorbance of control cells) × 100.

### Rat model for LAB safety evaluation

At the Animal Health Research Institute in Egypt's Laboratory Animal House, 20 healthy mature Sprague Dawley albino male rats weighting 120 ± 5 g and aged 8 weeks were purchased and accommodated for one week before conducting the experiment. The animals were kept in metal cages under hygienic conditions of 14 h/10 h light/dark cycles, a temperature of 24 ± 4 °C, and a relative humidity of 60 ± 10%. The rats were divided randomly into five equal groups: the first group served as the control and was fed with 1 mL of PBS, the second, third, and fourth groups were fed with 10^9^ CFU/mL of the *Enterococcus faecium* IS03 strain, *Enterococcus lactis* IS05 strain, *Enterococcus massiliensis* IS06 strain, and *Lactiplantibacillus plantarum* IS07 strain, respectively. Each group contained four rats. The sample size was calculated by power analysis using the G*power program^43^. All the researchers were aware of the group allocation at the different stages of the experiment. The trial has been running for three weeks, and all treatments have been received day after day. Ad libitum food and drink were provided before and during the experiment period. Loss of weight exceeding 20% of normal body weight was the humane endpoint for this study. There was no exclusion of any experimental group, any animals, any experimental units, or data points in this study. At the end of the experiment, all groups were sacrificed by decapitation under general anesthesia using a combination of ketamine (45 mg/kg) and xylazine (10 mg/kg) anesthetic agents. All experimental protocols were approved by the research ethics committee (REC) for experimental and animal studies at Cairo University, Egypt (approval number: CU II F 8 23). REC for experimental and animal studies regulated the handling and housing of the rats in compliance with institutional policies for laboratory animal use and care.

#### Biochemical determination

Immediately after drawing fresh blood samples, the sera were collected by centrifuging at 3000 rpm for 15 min. The alanine aminotransferase (ALT), aspartate aminotransferase (AST), alkaline phosphatase (ALP), uric acid, and creatinine concentrations were determined using the clear supernatant serum that had been aspirated into dry sterile vials and stored at -20 °C. ALT and AST levels were measured using a calorimetric method as previously reported^44,45^. Alkaline phosphatase (ALP) was determined using a previously described method^46^. Uric acid and creatinine were measured using a calorimetry method^47,48^.

Liver, kidney, and spleen samples were sliced and stored at -80 °C for further analysis of catalase activity (CAT), reduced glutathione (GSH), and superoxide dismutase (SOD). Superoxide dismutase (SOD) and reduced glutathione (GSH) were detected in the liver, kidney, and spleen as described previously^49,50^.

### Statistical analysis

Graphs and statistical analysis were conducted using R (version 3.2.5) and Adobe illustrator CC 2015. Student’s t test and the Mann‒Whitney–Wilcoxon test (nonparametric) were used to test the significance of comparisons. The variations were considered significant when the *p* value was ≤ 0.05.

## Results

### Isolation of LAB and in vitro safety evaluation

To obtain multiple LAB isolates to test their probiotic potentials, we isolated 100 bacterial isolates from eight fermented food sources on MRS agar media (Fig. 1). Only 19 isolates (5 isolates from soybean, 5 isolates from soy sausage, 3 isolates from plant-based luncheon meat, 3 isolates from pickles, and 3 isolates from corned beef) exhibited LAB properties, as they are Gram-positive, catalase-negative, and lactose-fermenters. To assess the in vitro safety of the isolated LAB, the isolates were submitted to the blood hemolysis test, where only 8 isolates (IS02, IS03, IS04, IS05, IS06, IS07, IS10, and IS18) showed no blood or γ-hemolysis (Fig. 1 and Table S1).

### Bile salt tolerance of the LAB isolates

**Figure 2:**
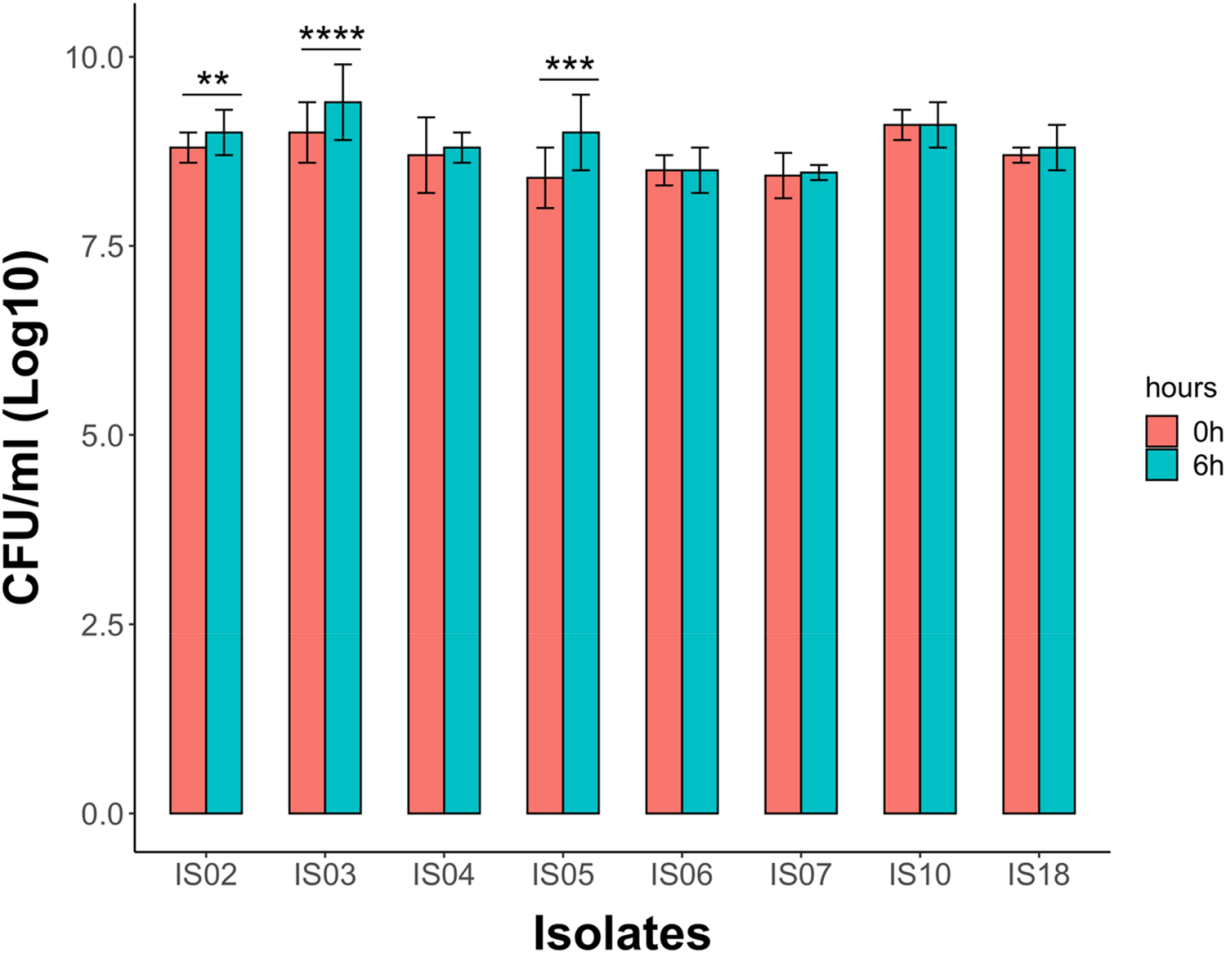
Bile salt tolerance of the selected LAB isolates for 6 h. Data are represented as the means of three replicate values ± standard error of the mean. **P < 0.01; ***P < 0.001; ****P < 0.0001.

One of the most important criteria for probiotic bacteria is their tolerance to bile salt to ameliorate their survivability in the GIT. Therefore, we tested the ability of the eight selected LAB isolates to tolerate 0.3% bile salt in MRS media for 6 h. All 8 isolates showed good bile salt tolerance, as their CFU counts were not affected by the presence of bile salt in the media for 6 h (Fig. 2). However, only three isolates (IS02, IS03, and IS05) showed a significant increase in their CFU counts (*P* < 0.01, 0.0001, and 0.001, respectively) after 6 h of growth in the presence of bile salt (Fig. 2).

### NaCl and phenol tolerance of the LAB isolates

**Figure 3:**
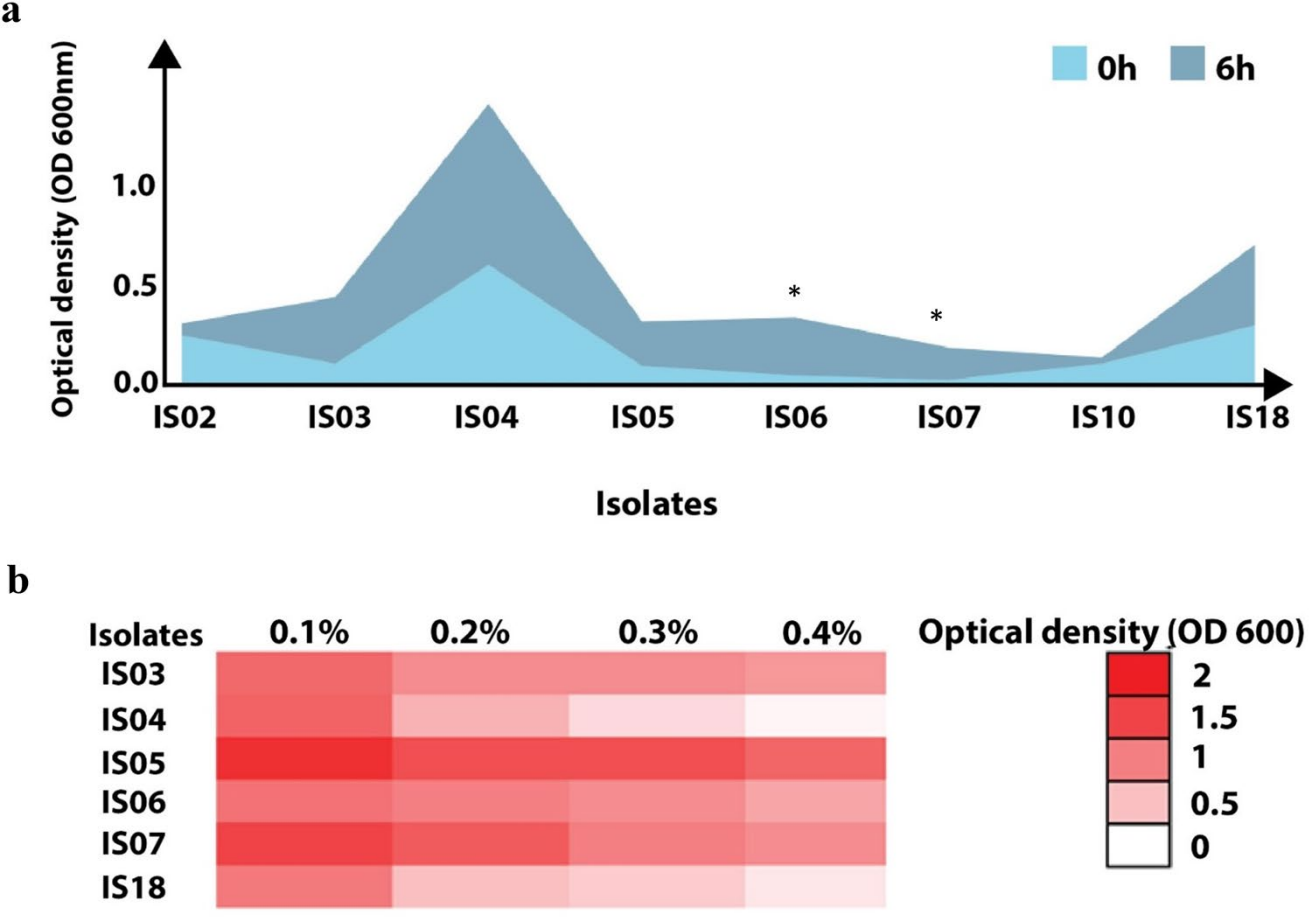
Tolerance of the selected LAB isolates to high concentrations of NaCl and phenol. (**a**) Tolerance to 8% (w/v) NaCl. (**b**) Tolerance to increasing concentrations of phenol (0.1–0.4%). The intensity of color in the heatmap reflects the OD of the bacterial growth at different concentrations. Data are represented as the means of three replicate values.

All 8 isolates were tested for their tolerance to high concentrations of NaCl and phenol. Only six isolates (IS03, IS04, IS05, IS06, and IS07, and IS18) were shown to have good tolerance to high NaCl levels (8%) after incubation for 6 h. There was a significant increase in the OD of only two isolates (IS06 and IS07) after 6 h of growth in the presence of 8% NaCl (*P* < 0.01). Conversely, the other isolates showed reduced growth at high NaCl concentrations (Fig. 3a and Table S2). Furthermore, the six selected isolates showed variable growth responses toward increasing concentrations of phenol (0.1–0.4%). Only four isolates (IS03, IS05, IS06, and IS07) showed good tolerance to high concentrations of phenol after growth for 24 h (Fig. 3b).

### Survival in simulated gastric and intestinal juice (simulated human digestive system)

**Figure 4:**
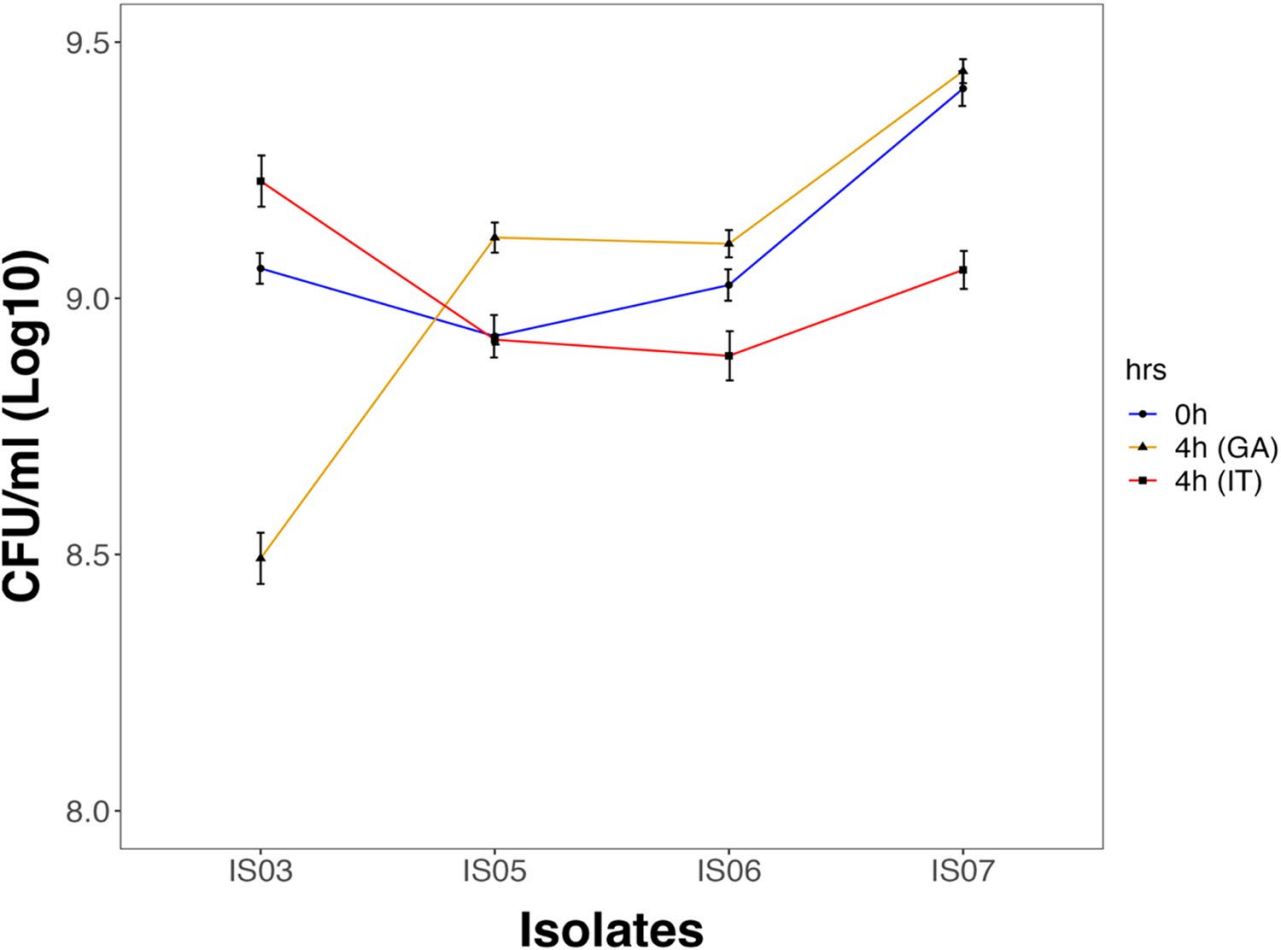
Survival of LAB isolates in simulated gastric (GA) and intestinal juices (IT) at 0 and 4 h of exposure. Data are represented as the means of three replicate values ± standard error of the mean.

The ability of LAB to survive both gastric and intestinal juices promotes their potential as probiotics. Therefore, we examined the survivability of the four selected LAB isolates in the presence of simulated gastric juice (pH 2) and simulated intestinal juice (pH 6.8) successively for 4 h. The four isolates survived both simulated gastric and intestinal juices for 4 h (Fig. 4). There was no significant difference in the CFU counts for all four isolates at 4 h compared to their CFU counts at zero time.

### Autoaggregation and coaggregation properties

All four isolates showed high autoaggregation percentages ranging from 78.37 ± 2.03 to 94.63 ± 4.07% after 2 h of growth (Table 1). Both IS05 and IS07 isolates exhibited significantly higher autoaggregation activities compared to IS03 and IS06 isolates. Consistently, all the isolates also showed high coaggregation capabilities with all tested pathogenic bacteria after 2 h of growth (Table 1). However, the coaggregation capacity differed among the selected LAB isolates, indicating that the coaggregation capacity is strain-specific and contingent upon the pathogenic bacteria being tested. IS06 exhibited the highest coaggregation capacity with *E. coli* O157:H (*P* < 0.05), *Staph. aureus*, and *B. cereus* (*P* < 0.01). While IS07 showed the highest coaggregation capacity with *L. monocytogenes* (*P* < 0.01), *Ps. aeruginosa*, and *Salm. enterica* serovar Typhimurium (*P* < 0.05).

**Table 1:** The aggregation capacities of the selected LAB isolates.

Isolates	*Autoaggregation %*	Co-aggregation %						
		*Escherichia coli* *O157: H7*	*Staphylococcus aureus*	*Methicillin-resistant Staphylococcus aureus*	*Bacillus cereus*	*Pseudomonas aeruginosa*	*Listeria monocytogenes*	*Salmonella enterica* serovar Typhimurium
IS03	78 ± 2.03	87.81 ± 0.05	91.98 ± 1.20	92.60 ± 0.08	94.43 ± 3.43	91.24 ± 2.73	89.61 ± 0.78	90.11 ± 3.27
IS05	94.63 ± 4.07***	91 ± 3.90	90.75 ± 2.35	81.46 ± 0.56	81.46 ± 1.26	88.21 ± 0.05	87.57 ± 2.98	91.64 ± 1.29
IS06	83.33 ± 3.06	92.79 ± 1.50*	92.14 ± 1.78	87.70 ± 2.21	97.17 ± 1.89**	91.24 ± 1.32	90.13 ± 0.09	91.12 ± 1.61
IS07	91.33 ± 3.21**	86.76 ± 0.45	88.92 ± 0.63	90.27 ± 4.40	88.57 ± 0.79	91.97 ± 2.14	93.46 ± 0.63**	94.25 ± 0.23*

Data are means of three replicate values ± standard error of the mean.

*P < 0.05, ** P < 0.01, *** *P* < 0.001.

### Cell surface hydrophobicity

To assess the cell surface hydrophobicity of the isolates, the bacterial affinity for xylene was measured. All four isolates exhibited a high adhesion percentage to xylene. IS07 showed the highest adhesion percentage (90.49 ± 2.67). While IS03, IS05, and IS07 showed 88.70 ± 3.05, 86.40 ± 2.43, and 87.72 ± 2.98 adhesion percentages, respectively.

### Antimicrobial activity of LAB isolates against pathogenic bacteria

The antagonistic effect of the four selected LAB isolates was examined against seven indicator pathogenic bacterial strains using an agar plug diffusion assay. All isolates were shown to exert antagonistic activities toward all pathogens tested in this study. The diameter of the inhibition zone against each pathogen varied from one LAB isolate to another, ranging from 8 ± 0.00 to 29 ± 0.46 mm (Table 2). IS03 displayed the highest inhibitory effect against *Salm. enterica* serovar Typhimurium (20 ± 0.43 mm) *and Ps. aeruginosa* (25 ± 0.23 mm). IS05, IS06, and IS07 displayed the highest inhibitory effect against *L. monocytogenes*.

**Table 2:** Distribution of the Inhibition zone diameters (mm) of LAB isolates against indicator pathogenic bacteria.

Isolates	Pathogen						
	*Listeria monocytogenes*	*Escherichia coli* O157: H7	*Bacillus cereus*	Methicillin-resistant *Staphylococcus aureus*	*Salmonella enterica* serovar Typhimurium	*Pseudomonas eruginosa*	*Staphylococcus aureus*
IS03	18 ± 0.13	15 ± 0.63	19 ± 0.57	20 ± 0.05	20 ± 0.43***	25 ± 0.23***	17 ± 0.36
IS05	25 ± 0.03	19 ± 0.20	20 ± 0.31	18 ± 0.19	8 ± 0.21	9 ± 0.03	15 ± 0.04
IS06	19 ± 0.32	18 ± 0.00	19 ± 0.12	18 ± 0.08	10 ± 0.00	9 ± 0.00	13 ± 0.18
IS07	29 ± 0.46**	18 ± 0.27	15 ± 0.28	20 ± 0.17	11 ± 0.09	8 ± 0.00	20 ± 0.20*

Data are means of three replicate values ± standard error of the mean.

*P < 0.05, ** P < 0.01, *** *P* < 0.001.

### Antibiotic susceptibility

The antibiotic resistance pattern of the selected LAB isolates to 12 commonly used antibiotics was examined using a disc diffusion assay (Table 3). In view of the results, all LAB isolates exhibited multiple antibiotic resistance to more than three antibiotics. All LAB isolates exhibited antibiotic resistance to oxacillin, ciprofloxacin, and vancomycin. In contrast, they were all susceptible to penicillin (except the IS06 isolate) and chloramphenicol. Both IS03 and IS05 isolates were resistant to nine antibiotics.

**Table 3:** The antibiotic resistance profile of LAB isolates toward 12 commonly used antibiotics using disk diffusion assay.

Isolates	CTR	P	AMP	GEN	OX	S	TE	VA	CIP	C	E	K
IS03	R	S	R	R	R	R	S	R	R	S	R	R
IS05	R	S	R	R	R	S	R	R	R	S	R	R
IS06	S	R	S	I	R	S	R	R	R	S	I	I
IS07	R	S	S	I	R	R	I	R	R	S	I	R

### Cytotoxicity against human colon cancer cell line

**Figure 5:**
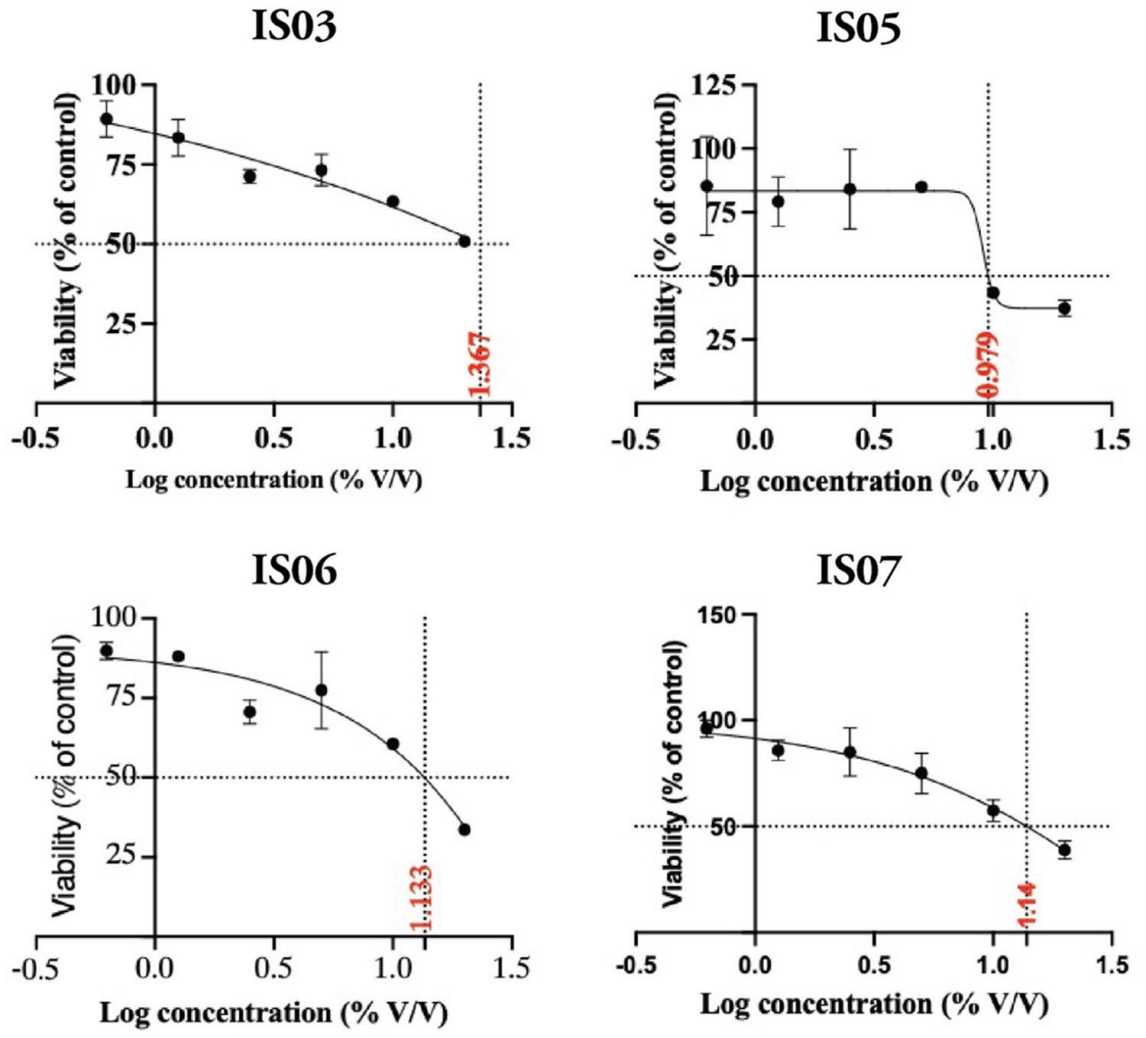
The cell proliferation activity of selected LAB strains on the Caco-2 cell line. The values represent the means ± standard error of four independent experiments.

**Figure 6:**
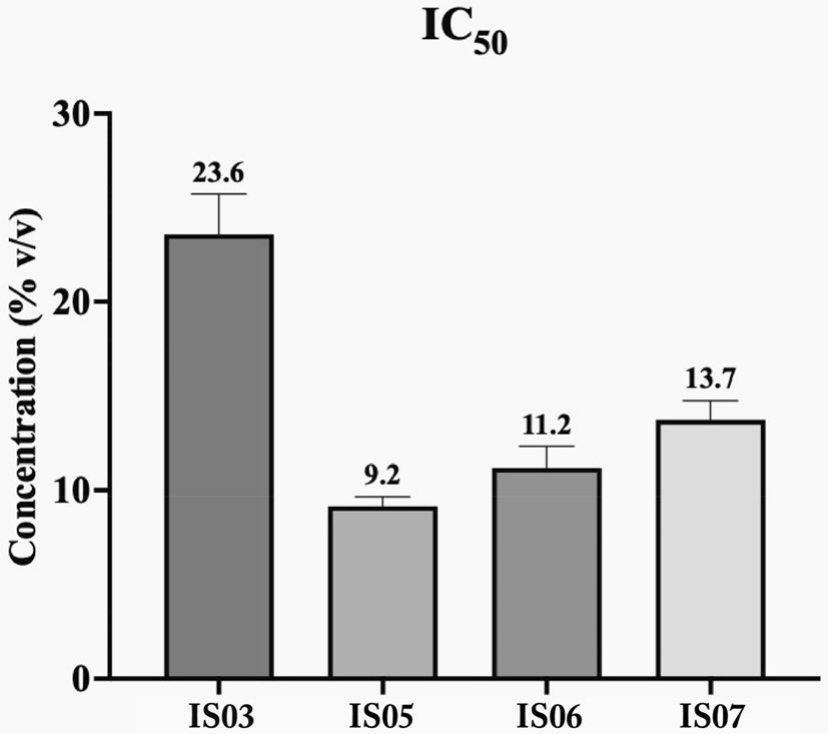
The IC50 of the tested LAB strains on the Caco-2 cell line. Data are mean values ± standard error of four independent experiments.

The cell proliferation activity of the four selected isolates was measured against the Caco-2 cell line using the MTT Cell Viability Assay. All isolates showed different cytotoxic effects. The cell proliferation activities of the LAB isolates and the IC_50_ are illustrated in Figs. 5 and 6, respectively. IS03 showed the least cytotoxic effect, with an IC_50_ value equal to 23.6% v/v. IS06 and IS07 showed moderate cytotoxicity, with IC_50_ values equal to 11.2 and 13.7% v/v, respectively. While IS05 showed the most cytotoxic effect, with the lowest IC_50_ value equal to 9.2% v/v.

### Molecular identification of the LAB isolates

**Figure 7:**
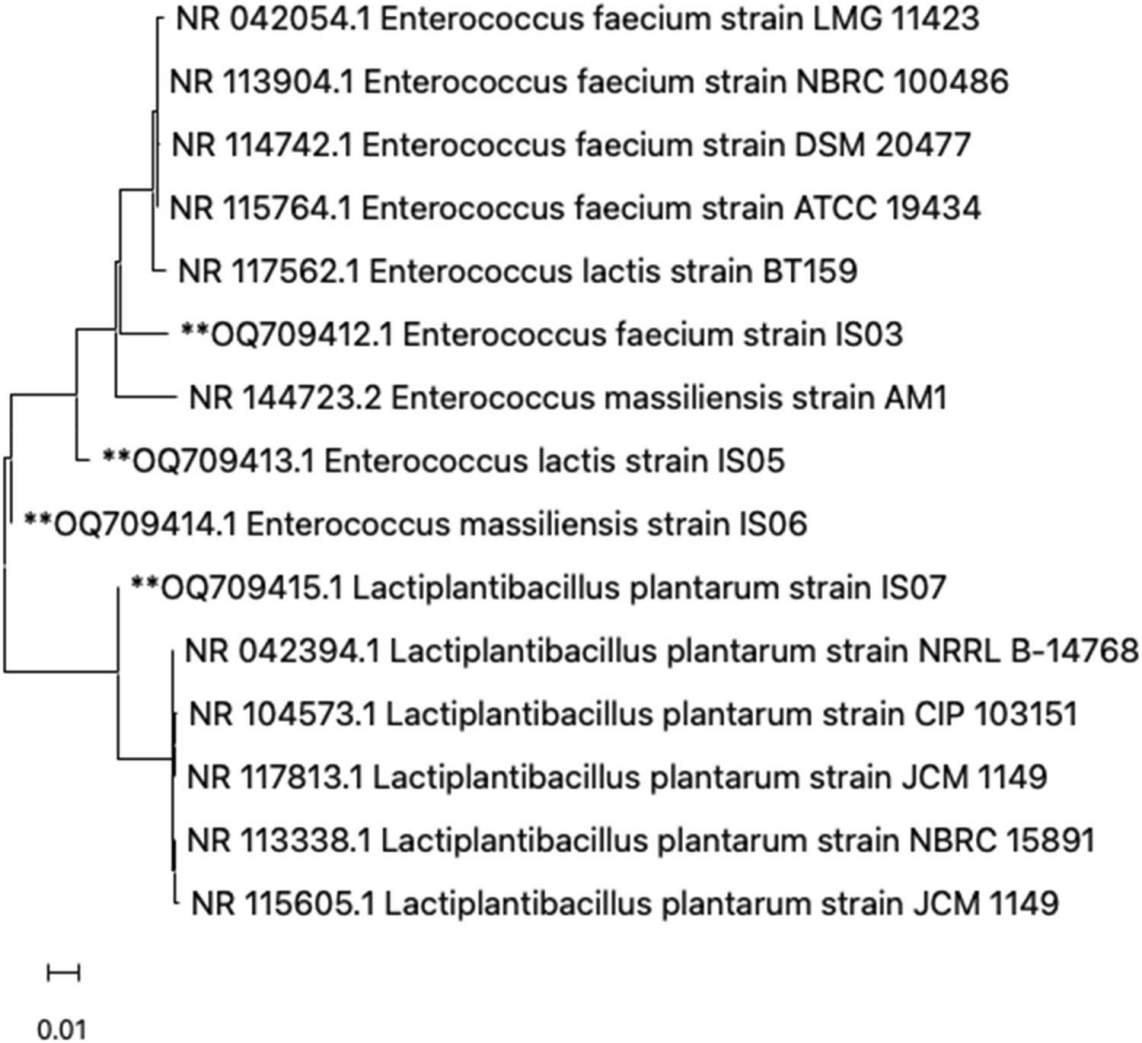
Phylogenetic tree of the potential LAB strains isolated and sequenced in this study (marked with **) based on their 16S rRNA sequences in relation to the closest strains.

The four selected LAB isolates shown to have potential probiotic properties were identified using 16S rRNA sequencing. The obtained 16S rRNA sequences were analyzed using the NCBI blast tool and deposited in NCBI GenBank (https://www.ncbi.nlm.nih.gov/genbank/). The accession numbers of IS03, IS05, IS06, and IS07 strains in NCBI GenBank are OQ709412, OQ709413, OQ709414, and OQ709415, respectively. The LAB strains were identified as *Enterococcus faecium* (IS03), *Enterococcus lactis* (IS05), *Enterococcus massiliensis* (IS06), and *Lactiplantibacillus plantarum* (IS07). Furthermore, Fig. 7 shows the phylogenetic tree of the sequenced LAB strains based on their 16S rRNA sequences in relation to the closest bacterial strains.

### In vivo safety evaluation of LAB strains

**Figure 8:**
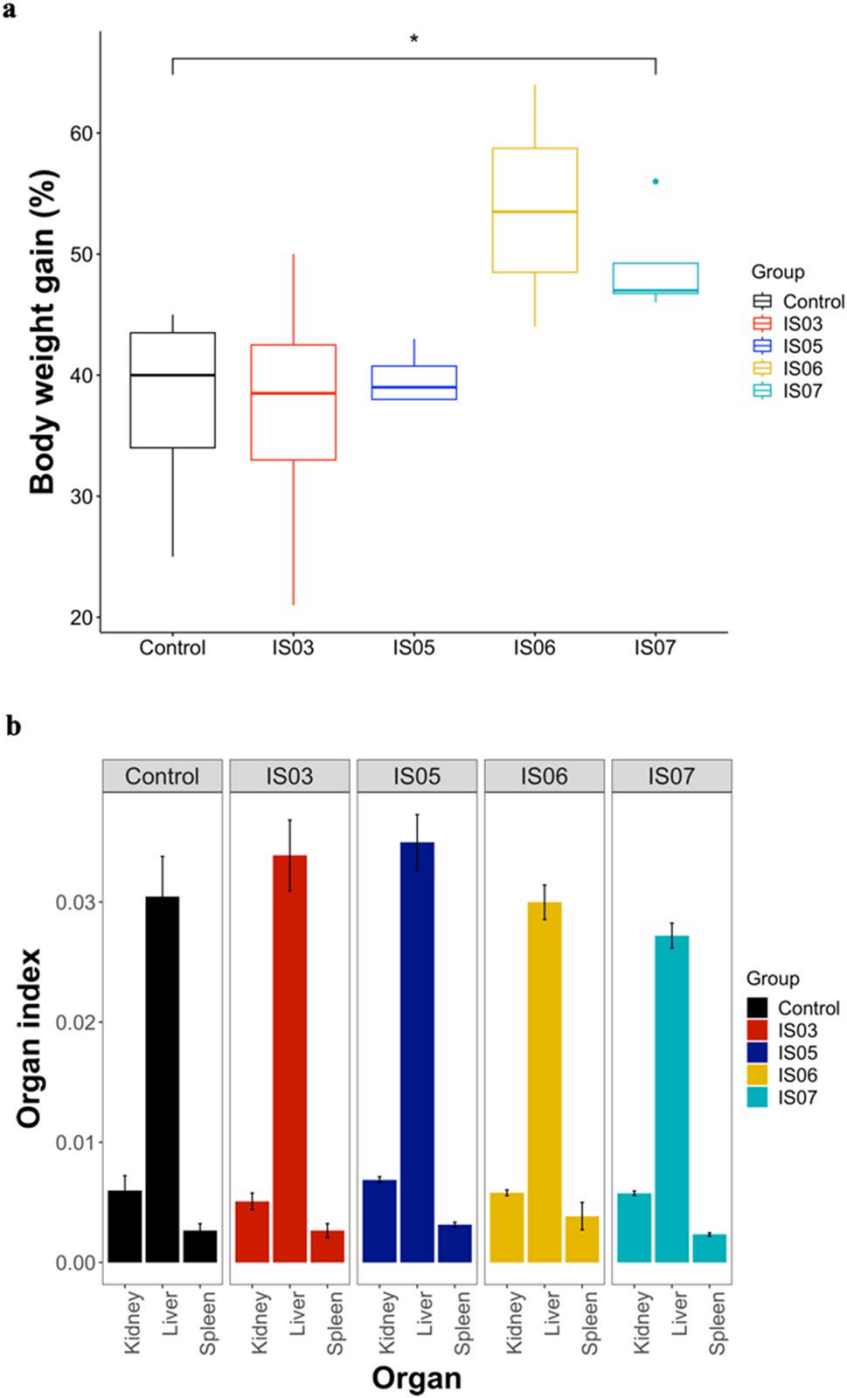
Impact of feeding rats with potential LAB probiotic strains on the body weight gain percentage and organ index of experimental rats. (**a**) Body weight gain. Data are represented as the median of four replicate values. *P < 0.05 in Mann‒Whitney-Wilcoxon test. (**b**) Organ index analysis of the kidney, liver, and spleen in all treated rat groups. Data are represented as the means of four replicate values ± standard error of the mean.

The safety of potential LAB probiotic strains was assessed in rats by feeding five rat groups with PBS as a control and the four LAB isolates. The body weight gain (BWG) was determined after 21 days of feeding with LAB strains. There was no significant difference observed in the BWG of rats treated with *Enterococcus faecium*, *Enterococcus lactis*, and *Enterococcus massiliensis*. However, there was a significant increase in the percentage of BWG in the rats treated with *Lactiplantibacillus plantarum* compared to the control group (*P* < 0.05) (Fig. 8a). Additionally, the organ index was determined for the kidney, liver, and spleen in all rat groups. Compared to the control group, there was no significant difference in the organ index in all rats treated with LAB strains (Fig. 8b). Furthermore, we determined liver and kidney functions by measuring the levels of indicator enzymes such as ALT, AST, and ALP. Uric acid and creatinine concentrations in the blood of all experimental rat groups were also determined as indicators of kidney function. According to the results, the levels were normal in all groups, and no significant changes in liver and kidney functions were observed in all LAB-treated rat groups compared to the control group (data not shown).

### Effect of LAB supplementation on antioxidant levels in rats

**Figure 9:**
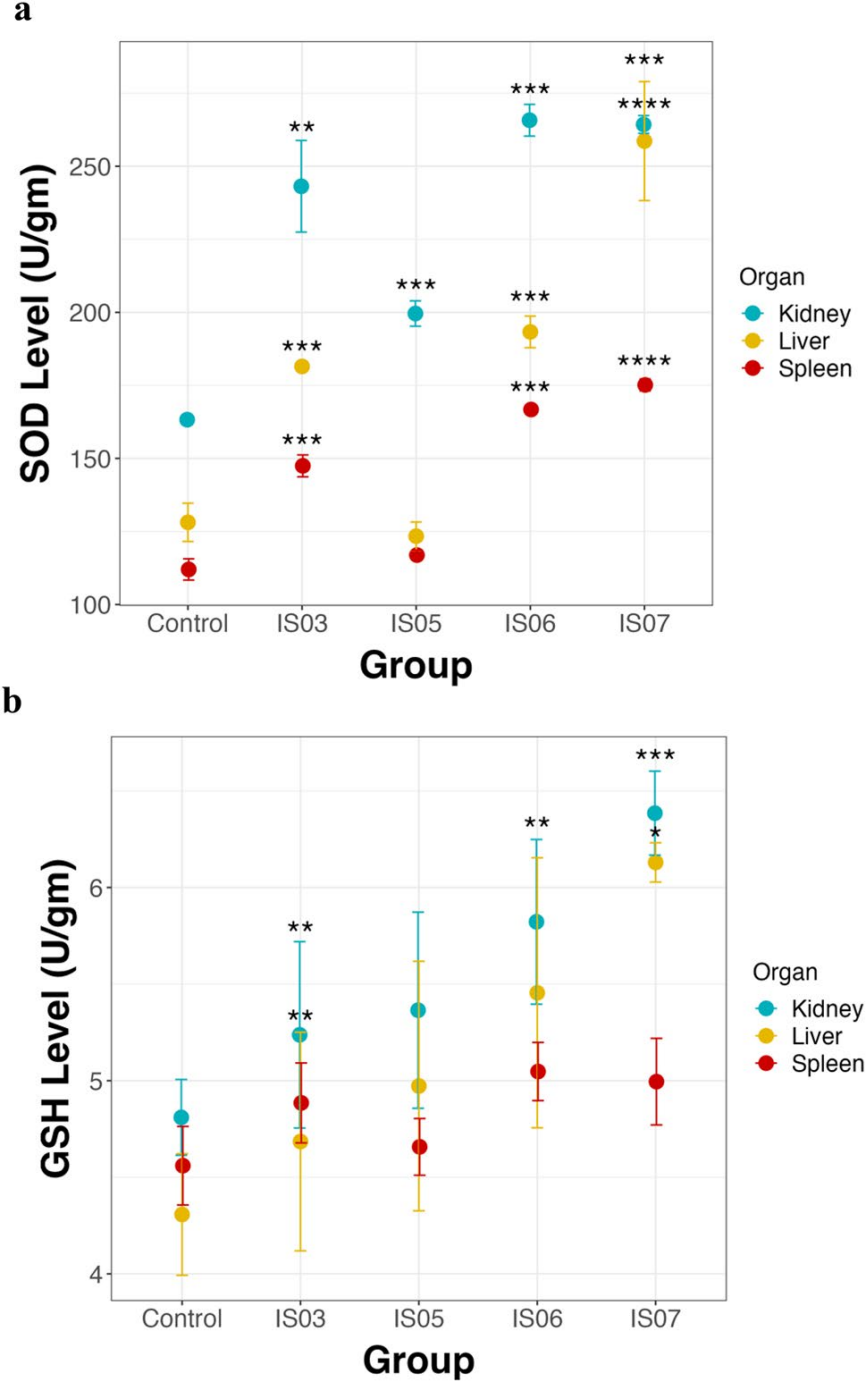
Antioxidant levels in the kidney, liver and spleen in all experimental rat groups. (**a**) SOD enzyme levels. (**b**) GSH levels. Data are represented as the means of four replicate values ± standard error of the mean. *P < 0.05, **P < 0.01; ***P < 0.001; ****P < 0.0001.

In this study, the levels of two key antioxidant compounds, SOD and GSH, was determined in the kidney, liver, and spleen. All experimental rat groups treated with LAB strains exhibited a significant increase in the levels of SOD enzyme in the kidney, liver, and spleen compared to the control group (Fig. 9a). On the other hand, only LAB-treated rats with *Enterococcus faecium* (IS03) and *Lactiplantibacillus plantarum* (IS07) exhibited a significant increase in GSH levels in the kidney and liver compared to the control group (Fig. 9b). The rat group treated with *Enterococcus massiliensis* (IS06) exhibited significantly increased GSH levels only in the kidney.

### Antioxidant status in the cellular extracts of LAB strains

To confirm the increased antioxidant activity of LAB strains shown in vivo, we measured the activity of both SOD and GSH-Px enzymes in the cellular extracts of LAB strains. The results showed that all the potential LAB probiotic strains exhibited high SOD enzyme activity. Only LAB strains IS05 and IS07 showed high GSH-Px enzyme activity (Fig. S1).

## Discussion

Fermented foods comprise a good reservoir of probiotic bacteria. Potential LAB probiotic strains, such as *Enterococcus faecium*, *Streptococci spp*., and *Lactobacillus plantarum,* have been extensively isolated from fermented food sources, including pickles, milk products, and fermented sausage^51,52^. The administration of LAB to humans and animals as an effective alternative to antibiotics and to confer multiple health benefits has recently attracted tremendous attention. Previous studies have shown that the application of probiotics to feed supplements is a promising strategy for providing a significant impact on livestock^53^. However, it is necessary to systemically characterize and identify these bacteria to ensure their safety and effectiveness. Consequently, the purpose of this study was to analyze and assess the probiotic properties, health benefits, and safety of LAB isolated from different fermented food sources in vitro and in vivo*.* In our research, we isolated and identified four LAB strains as potential probiotics from fermented food sources. They were identified as *Enterococcus faecium*, *Enterococcus lactis, Enterococcus massiliensis,* and *Lactiplantibacillus plantarum. Enterococcus faecium and Enterococcus lactis* strains were previously isolated from soy milk^54^ and found in high numbers in fermented cheese and sausage^55^.* Ent. faecium* strains were reported in previous studies to have probiotic properties and to confer positive health effects^56^. *Ent. lactis* strains were also reported to be safe and to have probiotic properties^57,58^. *Lactiplantibacillus plantarum* strains have been isolated previously from different food products and were shown to have probiotic properties^59,60^. Furthermore, *Ent. massiliensis* was reported exclusively in this study as a potential probiotic strain with different applications.

The capacity of LAB strains to tolerate bile salt in the duodenum and extreme pH conditions in the stomach is one of the key factors affecting their survival rate in the host. According to our results, LAB strains exhibited varying levels of bile salt resistance, which may be attributed to the expression of proteins related to bile salt resistance^61^. In this study, the viability of both *Ent. faecium* IS03 and *Ent. lactis* IS05 strains was significantly increased after exposure to bile salts for six hours. *Enterococcus* bacteria are the indigenous microflora inhabiting the GI tract of humans^62^ and possess different benefits in the dairy industry. Therefore, *Enterococcus* strains are expected to survive extreme GI conditions. Furthermore, our LAB strains showed good survivability in simulated gastric juice at pH 2 and simulated intestinal juice at pH 6.8. These findings are probably due to the presence of strain- or species-specific tolerance mechanisms^63^ with potential resistance-producing bacterial proteins^64^.

In this study, only six LAB isolates showed high tolerance to 8% NaCl, and only two isolates showed a significantly high tolerance. These two isolates were isolated from soy sausage and pickles. This is in agreement with previous studies showing that LAB isolated from dairy products, pickles, chickens, and meats can tolerate high NaCl concentrations^65–67^. These results represent desirable characteristics for potential LAB probiotics that could promote bacterial growth and the production of advantageous metabolites. In addition, the ability of LAB strains to survive high levels of NaCl is relevant to industrial and food preservation applications. Moreover, only four LAB strains demonstrated good survival at increasing concentrations of phenol. Phenol is a toxic metabolite secreted by microbes in the GIT when some amino acids are deaminated^68^. Consequently, potential probiotic strains should have the capacity to tolerate phenolic conditions to survive under GIT conditions. LAB strains were reported previously to exhibit varying degrees of phenol tolerance^69^.

Surface colonization of LAB strains in the GIT due to autoaggregation, coaggregation, and hydrophobicity is one of the key factors affecting probiotic evaluation. The autoaggregation feature enables microorganisms of the same species to adhere to the intestinal mucosa^70^. In contrast, coaggregation allows intracellular adhesion between various strains of microorganisms, which is dependent on the capacity of strains to interact with pathogens. Therefore, aggregation could play a vital role in the host’s defense mechanisms against infections^71^. In this study, the four LAB strains were observed to have efficient autoaggregation and coaggregation capabilities after only 2 h of growth. This could be due to the intricate interactions among bacterial cell-surface proteins or other secreted metabolites. The aggregation phenotype is mainly dependent on the surrounding environment and potential internal factors^71^. Therefore, it is necessary to identify the factors involved in the aggregation phenotype of these strains. Additionally, LAB exhibiting aggregation-promoting factors may promote the elimination of pathogens by maintaining the balance of gut microbial interactions and the coaggregation mechanism. Cell-surface hydrophobicity is another major factor for evaluating the probiotic properties of LAB strains. The ability of probiotic strains to adhere to enterocyte cells is directly measured by their hydrophobicity^72^, which is a favorable property of probiotics. All four LAB strains examined in this work showed high cell-surface hydrophobicity, with values higher than 85%. This suggests that the adherence capability of these LAB strains to the intestinal layer would be very efficient. In accordance with a previous report^73^, a hydrophobicity of 40% or above is used as a standard for selecting potential LAB probiotics. Thus, the high autoaggregation, coaggregation, and hydrophobicity potentials of the LAB strains isolated in this study can aid their presence and persistence in the human and animal intestinal tract to improve intestinal health.

A substantial amount of evidence suggests that several probiotic bacteria can exhibit antagonistic activity against intestinal pathogenic microbes^74–76^. As more considerable attention has been given to the dissemination of antibiotic resistance, antimicrobial activity against pathogenic microbes has garnered extensive concern^77^. Putative probiotics should harbor antimicrobial properties against both Gram-positive and Gram-negative bacteria. All four LAB strains showed high antimicrobial activities against the pathogenic bacteria tested in this study. In fact, the antimicrobial activity is relevant to coaggregation activity^78^. Probiotic bacteria produce antimicrobial substances during their intimate contact with pathogenic bacteria to eliminate pathogens^79^.

Potential probiotics are characterized by harboring intrinsic and non-transmissible genetic factors that aid them in developing antibiotic resistance to different antibiotics without serving as a reservoir of antibiotic resistance genes^80^. It is believed that antibiotic resistance in probiotics is essential for the survival of antibiotics in the GI tract^81^. The intestinal flora can be restored after antibiotic treatment due to the presence of probiotics with intrinsic resistance^82^. The antibiotic resistance of LAB strains against different types of antibiotics is a major criterion for their safety assessment as probiotics^83^. All four LAB strains in this study showed antibiotic resistance to oxacillin, ciprofloxacin, and vancomycin. On the other hand, they showed susceptibility to penicillin and chloramphenicol. This is in accordance with previous studies showing that most of the LAB strains isolated from the intestine of pigs and poultry were resistant to oxacillin, ciprofloxacin, and vancomycin and susceptible to penicillin and chloramphenicol^65,78^. Vancomycin-resistant LAB strains were also isolated from fermented cream^82^.

One of the most promising studied areas of the probiotics’ health-promoting effects is their anticancer activity^84,85^. Colorectal cancer (CRC) has recently emerged as one of the major diseases linked to gastrointestinal issues. According to epidemiologic studies, this cancer type constitutes up to 9% of all fatal cancers^86^. Intriguingly, certain probiotic bacteria can reduce the risk of CRC incidence^87^. There are different anti-CRC mechanisms employed/deployed by probiotics, including anti-inflammatory responses, DNA damage reduction, altering the enzyme activity of intestinal microflora, competitive colonization, and apoptosis induction^88,89^. In this study, all four LAB strains inhibited the proliferation of the colon cancer cell line (Caco-2) in a dose-dependent manner. The *Ent. lactis* IS05 strain showed the highest anti-colon cancer activity, while *Ent. massiliensis* and *Lactiplantibacillus plantarum* showed moderate anti-colon cancer activity. *Lactobacillus* strains were previously shown to inhibit the proliferation of cancerous cells, especially the Caco-2 cell line, by initiating the apoptotic process^90^. Additionally, both *Ent. faecium and Ent. lactis* were shown to have an antiproliferative effect against the Caco-2 cell line^91,92^. *Ent. massiliensis* was reported here for the first time to have cytotoxic effects against the cancer cell line Caco-2.

Although LAB as probiotics have long been considered safe for use, the pathogenicity and virulence of LAB strains must be assessed. None of the four selected LAB strains showed hemolysis of the sheep blood erythrocytes, indicating their non-virulent nature. Thus, these strains can be regarded as promising candidates for safe probiotics. Additionally, the safety profile of the four LAB strains was assessed by testing their effects on the overall health status of rats. No abnormal behavior, body weight loss, or changes in organ indices were detected after 21 days of feeding rats with the LAB strains. Also, the levels of indicator enzymes for liver and kidney functions were normal in rats supplemented with the LAB strains. This indicates that these four strains have no adverse effects on the general health of rats. Moreover, the growth performance of rats supplemented with the *Lactiplantibacillus plantarum* strain significantly increased. These findings support the evidence that LAB supplementation may benefit the health and well-being of animals.

Reactive oxygen species (ROS) can be produced in the human body due to different physiological processes^93^. The accumulation of ROS such as superoxide, hydrogen peroxide, and hydroxyl free radicals leads to severe damage to macromolecules such as DNA, lipids, and proteins. This would result in organ dysfunction^94^. To detoxify and scavenge free radicals, LAB can produce antioxidant enzymes. In addition, LAB produce exopolysaccharides^22,95^, lipoteichoic acid, and cell-surface proteins^96^ that may be responsible for their antioxidant activity. The levels of ROS can be reduced by the activity of antioxidant enzymes such as SOD, CAT, and the glutathione/glutathione reductase system. In our study, all four LAB strains demonstrated significantly high levels of SOD antioxidant enzyme in the liver, kidney, and spleen of the rats. However, all LAB strains except *Ent. lactis* IS05 showed high GSH levels in only the kidney and liver tissues of the rats. This is in line with previous studies showing that *Lactiplantibacillus plantarum* dramatically increased the levels of both SOD and GSH-Px enzymes in the liver of mice^97^. *Additionally, Lactiplantibacillus plantarum* isolated from raw fermented meat products showed high antioxidant activity^98^. *Enterococcus* species isolated from dairy and meat products were shown to exhibit high antioxidant activity^99^. These results indicate that LAB supplementation in humans and animals would enhance antioxidant activities, and LAB could be used in food processing as natural antioxidants instead of chemical antioxidants.

## Conclusion

This study showed that exploring new probiotics in fermented food products is a paramount avenue to attain a good supply of probiotic LAB strains with different health benefits. LAB isolated from different fermented food products are considered safe and harbor probiotic properties. Four LAB strains were shown in this study to tolerate bile salt and endure extreme conditions in the GIT. They were also shown to have autoaggregation, coaggregation, and hydrophobicity properties. In addition, these LAB strains were shown to have anti-pathogen, anti-colon cancer, and antioxidant activities. *Enterococcus massiliensis* is a novel probiotic strain isolated from soy sausage with different benefits. These findings indicate that fermented food products are a good source of probiotic bacteria.

### Supplementary Information

Supplementary Information.

## Data Availability

The datasets generated during and/or analyzed during the current study are available from the corresponding author on reasonable request.
