# Supplementary material for: Probiotic potentials of lactic acid bacteria isolated from Egyptian fermented food
Source: Sci Rep. 2023 Oct 3;13:16601. doi: 10.1038/s41598-023-43752-0 (PMC10547719; doi:10.1038/s41598-023-43752-0)
Supplement: Supplementary file 1 — Supplementary Information. [file 41598_2023_43752_MOESM1_ESM.pdf]

Probiotic potentials of lactic acid bacteria isolated from Egyptian fermented food

Fatma I. Abdel Tawab[4†], Menna H. Abd El kadr[3†], Amany M. Sultan[2], Ehdaa O. Hamed[2], Ayatollah S. El-Zayat[1], Marwa N. Ahmed[1*]

1. Department of Microbiology, Faculty of Agriculture, Cairo University, Giza, Egypt.
2. Department of Biochemistry, Toxicology Unit, Animal Health Research Institute, Agricultural Research Center, Giza Egypt.
3. Microbiology Lab, Research Park, Faculty of Agriculture, Cairo University, Giza, Egypt.
4. Oil Crops Biotechnology Lab, Agricultural Genetic Engineering Institute, Agricultural Research Center, Giza Egypt.

Running title: Fermented food products as a potential source of probiotics

[†]These authors contributed equally to this work and share first authorship.

*Corresponding author:

Marwa N. Ahmed, PhD

Lecturer

Department of Microbiology

Faculty of Agriculture

Cairo University

El-Gamaa Street

12613

Giza, Egypt

marwa.nabil@agr.cu.edu.eg

https://orcid.org/0009-0009-3685-6859

Table S1 List of the selected eight LAB isolates and the fermented food sources for each isolate

**Isolates**	**Fermented food source**
IS02	Soybean
IS03	Soybean
IS04	Soybean
IS05	Plant-based luncheon meat
IS06	Soy sausage
IS07	Pickles
IS10	Soy sausage
IS18	Pickles

Table S2 Tolerance of the selected LAB isolates to 8% NaCl

	Optical density (OD 600)	
**Isolates**	**0h**	**6h**
**IS02**	0.25 ± 0.01	0.06 ± 0.00
**IS03**	0.11 ± 0.03	0.33 ± 0.02
**IS04**	0.60 ± 0.02	0.80 ± 0.04
**IS05**	0.10 ± 0.03	0.23 ± 0.03
**IS06**	0.05 ± 0.02	0.29 ± 0.03**
**IS07**	0.03 ± 0.01	0.15 ± 0.00**
**IS10**	0.11 ± 0.01	0.03 ± 0.02
**IS18**	0.30 ± 0.05	0.40 ± 0.01

Data are represented in means of three replicates ± standard error**.** **$P < 0.01$

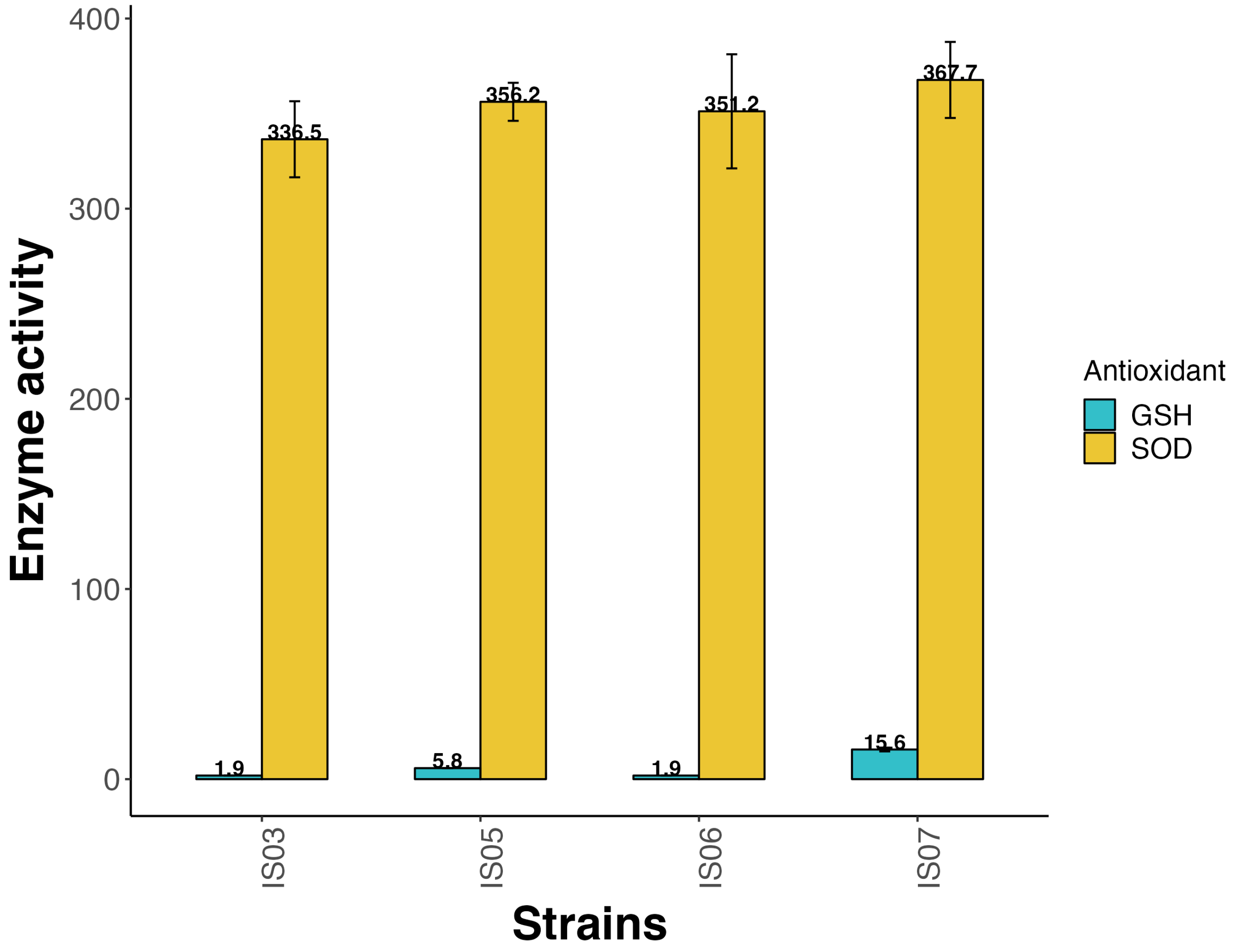

Ffig. S1 Antioxidant levels in the cellular extract of LAB strains. Data are represented in means of three replicate values ± standard error of the mean
